# NF-κB signaling pathway in osteosarcoma: from signaling networks to targeted therapy

**DOI:** 10.3389/fonc.2025.1565760

**Published:** 2025-07-25

**Authors:** Juandi Xue, Xiao-ran Yang, Li Wang

**Affiliations:** The First People’s Hospital of Lanzhou City, Lanzhou, Gansu, China

**Keywords:** osteosarcoma (OS), NF-κB signaling pathway, targeted therapy, chemotherapy, mechanism

## Abstract

Osteosarcoma (OS) is the most common primary malignant bone tumor that frequently occurs in children and adolescents. Although neoadjuvant chemotherapy has shown efficacy for OS, the long-term survival rate of OS patients remains low, highlighting the necessity of seeking more effective treatment methods. In cancer cells, abnormal activation of signaling pathways can widely affect cell activity from growth and proliferation to apoptosis, invasion and metastasis. As a highly complex and unique signal transduction pathway, the NF-κB pathway is involved in various physiological and pathological processes. In the field of oncology, the abnormal activation of the NF-κB pathway is closely related to the occurrence, development, metastasis and drug resistance of tumors, and is regarded as an important potential target for tumor treatment. Studies have confirmed that the NF-κB signaling pathway is an important driver of osteosarcoma. Abnormal activation of this pathway can promote the proliferation of osteosarcoma cells, inhibit apoptosis, enhance migration and invasion abilities, and immune escape processes. While inhibition of NF-κB pathway can effectively inhibit or reverse the above pathological processes. In this review, we summarized the role and mechanism of the NF-κB pathway in OS, discussed the therapeutic significance of targeting this pathway for OS, as well as the current insufficient research and problems to be solved regarding this pathway in OS. This review is helpful for us to understand the role of NF-κB on OS, and provides a theoretical basis for targeting the NF-κB pathway as a therapeutic target for OS and developing new therapeutic strategies.

## 1 Introduction

Osteosarcoma (OS) is the most common primary malignant bone tumor that frequently occurs in children and adolescents (1), and its biological behavior is significantly characterized by invasive proliferation and a tendency of early metastasis (2, 3). At present, the treatment of OS mainly includes multi-scientific and multi-modal treatments such as surgery, radiotherapy, neoadjuvant chemotherapy and postoperative adjuvant chemotherapy. However, their effects are not satisfactory, and there are still high risks of metastasis, recurrence and chemotherapy resistance after treatment (4). Data show that the five-year survival rate of patients with metastatic osteosarcoma is generally lower than 25% (5). Moreover, the progression mechanism of osteosarcoma is complex. Its molecular pathological mechanism involves abnormal regulation of multiple signaling pathways, and the key driver genes have not been fully clarified yet. Based on the current treatment predicament, there is an urgent need for new intervention methods and strategies to improve the prognosis and quality of life of patients with osteosarcoma.

Although the pathogenic mechanism of OS has not been fully clarified, existing studies have revealed that it is closely related to the dysregulation of multi-signaling pathway networks, among which the abnormal activation of the NF-κB signaling axis is particularly crucial (6, 7). As a core molecular hub regulating immune responses, inflammatory responses and apoptosis (3, 8–10), this signal network can regulate core biological processes such as cell cycle progress, apoptosis and stress responses (8, 11, 12). Based on this, the NF-κB signaling pathway can be involved in the occurrence, development and pathological mechanisms of various diseases, such as myocarditis, spinal cord injury, degenerative changes of intervertebral discs, traumatic brain injury and other pathological processes of various diseases (13–17). Furthermore, abnormal NF-κB signaling has been observed in many human diseases and is often a contributing factor, including autoimmune diseases, chronic inflammatory diseases and cancers (18–20). Studies have shown that the NF-κB signaling pathway, as a key carcinogenic regulatory network, plays an important role in the occurrence and development of various malignant tumors in humans, such as solid tumors like prostate cancer, colorectal cancer, bladder cancer, breast cancer and osteosarcoma (21–25). This pathway plays a dual role in tumor evolution: it not only maintains the survival advantage of malignant cells but also promotes tumor progression by enhancing the ability of invasion and metastasis and inducing angiogenesis (26, 27). It is notable that the latest research reveals that the activation of this pathway is not only directly related to the epithelial-mesenchymal transition (EMT) of tumor cells, but also further exacerbates the malignant phenotype of tumors by reshaping the immunomodulatory and inflammatory response networks in the tumor microenvironment. Studies have shown that the NF-κB signaling axis is in a persistently activated state in osteosarcoma, and its abnormal activity significantly accelerates the pathological process of OS by driving the malignant proliferation of tumor cells (28). It is particularly notable that the multi-dimensional regulatory function of this pathway has extended to the core field of tumor biology: in addition to participating in the cell cycle disorder and apoptotic escape of OS cells, it is more deeply involved in the formation mechanism of key malignant phenotypes such as chemotherapy resistance and distant metastasis (29, 30). In recent years, breakthroughs in molecular targeting research have confirmed that the NF-κB pathway is an important molecular mechanism in the occurrence and development of OS. Intervention strategies targeting this signaling network have shown the potential to improve prognosis by precisely blocking the cascade reaction of OS onset (31).

In this article, we summarize the structural characteristics of the NF-κB signaling pathway, focus on analyzing its dynamic regulatory network in the remodeling of the OS microenvironment, phenotypic transformation and treatment resistance, and explore the synergistic treatment strategy based on the multi-target characteristics of the pathway. The limitations of the current research deficiencies were discussed, and corresponding strategies were proposed, aiming to provide a theoretical framework and transformational perspective for the development of new precision diagnosis and treatment plans for OS.

## 2 NF-κB signaling pathway

### 2.1 Overview of NF-κB pathway

**Figure 1:**
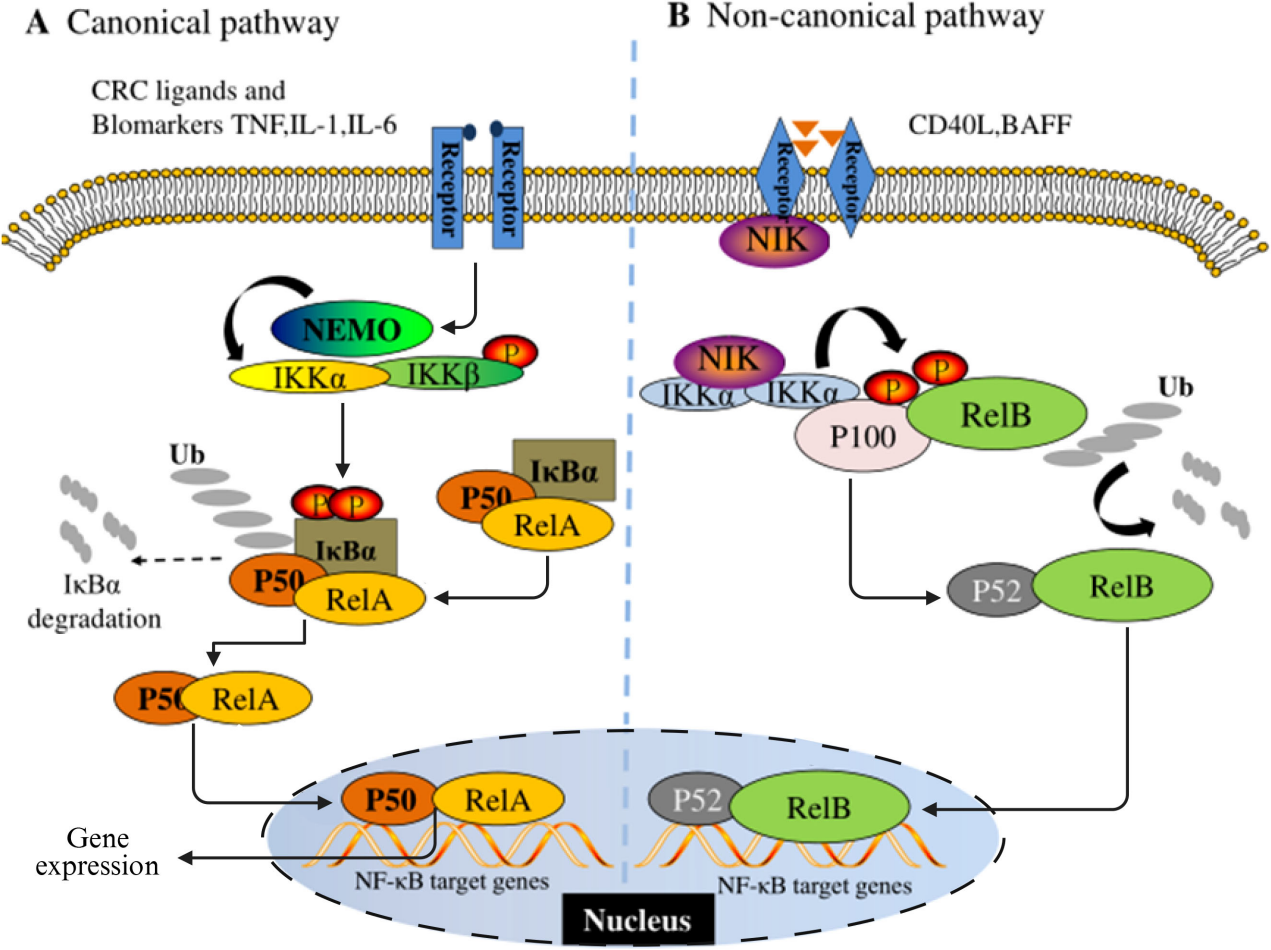
Schematic diagram of the NF-κB signaling pathway. **(A)** is the classical pathway and **(B)** is the non-classical pathway.

As an evolutionarily conserved member of the Rel oncogene family, NF-κB was initially described as a B-cell-specific factor that specifically binds to DNA sites in the intron enhancer region of the immunoglobulin κ light chain gene (32). In mammalian systems, this family consists of five structural subtypes: p65 (RelA), RelB, c-Rel, p50/p105 and p52/p100. Each member achieves functional regulation through anchor protein repeat domains. As the dimerization transcription factor prototype of the Rel protein family, NF-κB constitutes a complex signal decoding system through the synergistic combination of different subunits. The molecular architecture feature of NF-κB protein is manifested as a Rel homologous domain (RH) composed of approximately 300 amino acids at its N-terminal. This functional module not only mediates dimerization formation but also undertakes the key molecular docking function of DNA-specific binding and the interaction of IκB inhibitors (33, 34). According to the differences in their structural characteristics, functions and biosynthetic pathways, the Rel protein family can be systematically divided into two functional subgroups: Class I members (p50/NF-κB1 and p52/NF-κB2) are produced through the proteolytic processing of precursor proteins; Members of Class II (p65/RelA, c-Rel, RelB and Fruit fly Rel homologous proteins) directly express the mature form, and the specific transcriptional activation domain (TAD) at the C-terminal endows them with the ability to directly regulate gene expression (35, 36). These two types of proteins, through dynamic dimerization combination, form signal transduction networks in the classical and non-classical pathways of the NF-κB signaling pathway respectively (**Figure 1**).

NF-κB dimer usually exists in the cytoplasm in an inactive state in most mammalian cells. The maintenance of this homeostasis is mainly attributed to its interaction with the inhibitory protein family IκB. The currently identified members of the IκB family include IκBα, IκBβ, IκBε, Bcl-3, and two precursor proteins p100 and p105. The common feature of these proteins is that they have anchor-protein-like repeating domains, mediating the binding and inhibition of NF-κB (37). NF-κB as a transcription factor widely present in eukaryotes, has a significant environmental dependence in its activation mechanism. The activation of NF-κB requires the release of molecules from its inhibitory Iκb, which is initiated by various stimuli and conditions related to immune function and development. Analyzed from the regulatory network level, the initiating factors of the NF-κB system have functional diversity characteristics and can mainly be classified into three categories: 1) Members of the cytokine family, such as tumor necrosis factor -α (TNF-α), interleukin-1 (IL-1), nuclear factor κB receptor activator ligand (RANKL), etc. 2) Pattern recognition receptor ligands, including bacterial lipopolysaccharide (LPS), Toll-like receptor (TLR) specific agonists, etc. 3) Immunomodulatory signaling molecules, such as CD40 ligand (CD40L), lymphotoxin, etc. (38). The activation pattern of the NF-κB signaling pathway has significant signal specificity. The activation pattern of the NF-κB signaling pathway has significant signal specificity. In the classic inflammation-related pathways, pro-inflammatory cytokines such as IL-1β preferentially induce the nuclear translocation of RelA/cRel heterodimer through receptor-mediated activation of the IκB kinase (IKK) complex (39). The core regulatory mechanism of this process relies on the phosphorylation modification of IκB inhibitory protein and its subsequent ubiquitination degradation (36, 40). From the perspective of functional genomics, NF-κB participates in multi-dimensional pathological processes by regulating the expression network of target genes. Its transcription products include pro-inflammatory cytokines (such as TNF-α, IL-6), matrix metalloproteinases (MMPs), anti-apoptotic proteins (Bcl-2 family), and cell adhesion molecules (VCAM-1/ICAM-1), etc. These molecules jointly constitute the microenvironment basis for tumorigenesis and development (41). It is worth noting that the osteosarcoma research model shows that compared with normal bone cells, the NF-κB binding level in human osteosarcoma cell culture is significantly increased, suggesting that its abnormal activation is correlated with the malignant phenotype (42). Furthermore, the clinicopathological evidence further reinforces this association. Tissue microarray analysis indicated that more than 75% of osteosarcoma samples presented positive nuclear characteristics of NF-κB signaling, and this activation status was significantly correlated with the prognosis of patients (43). These multi-dimensional pieces of evidence systematically explain the pivotal role of NF-κB in the occurrence, progression and treatment resistance of osteosarcoma, ranging from molecular mechanisms to clinical phenotypes.

### 2.2 Effect of genetic changes on abnormal activation of NF-kB pathway

Genetic alterations (mutations in the TP53, MYC, and PTEN genes) have been found to affect the activation of NF-κB in OS. TP53 gene is an important tumor suppressor gene, and its mutation is closely related to the dysfunction of NF-κB signaling pathway (44). Normally, p53 inhibits the activation of the NF-κB signaling pathway through a variety of mechanisms. However, when TP53 is mutated, p53 protein loses its inhibition of NF-κB, resulting in overactivation of the NF-κB signaling pathway (45). For example, in some osteosarcoma cells, mutation of the TP53 gene significantly enhances the activity of NF-κB, promoting the proliferation, survival, and metastasis of tumor cells. At the same time, mutated p53 may act synergistically with NF-κB to regulate some common target genes and further promote tumor development (46).

MYC gene is a proto-oncogene whose amplification or overexpression is common in a variety of tumors and also leads to abnormal activation of the NF-κB signaling pathway (47). MYC can indirectly activate NF-κB through a variety of pathways. On the one hand, MYC promotes cell proliferation and metabolism, and increases the production of intracellular reactive oxygen species (ROS), which can activate IKK complex and then activate NF-κB signaling pathway (48). On the other hand, MYC can up-regulate the expression of some genes related to NF-κB activation, such as TRAF2, RIP1, etc., and enhance the activity of NF-κB signaling pathway. In osteosarcoma, MYC overexpression is positively correlated with NF-κB activation, which together promote tumor cell proliferation, invasion, and metastasis. In addition, MYC and NF-κB can also regulate each other’s target genes, forming a complex regulatory network to further promote the occurrence and development of osteosarcoma (49). PTEN gene is a tumor suppressor gene that encodes a protein with phosphatase activity that negatively regulates the phosphatidylinositol-3 kinase (PI3K)/AKT signaling pathway. Normally, PTEN indirectly inhibits the activation of NF-κB by inhibiting the PI3K/AKT signaling pathway (50). When PTEN is mutated or deleted, the PI3K/AKT signaling pathway is over-activated, and AKT phosphorylates and activates the IKK complex, thereby activating the NF-κB signaling pathway (43). In osteosarcoma, the absence or low expression of PTEN leads to sustained activation of the NF-κB signaling pathway, which promotes tumor cell proliferation, survival, and migration. At the same time, the activation of NF-κB can further up-regulate the expression of some genes related to tumor cell proliferation and metastasis, such as MMP-9 and VEGF, and enhance the malignancy of osteosarcoma (50).

## 3 Role of NF-κB pathway in OS

**Figure 2:**
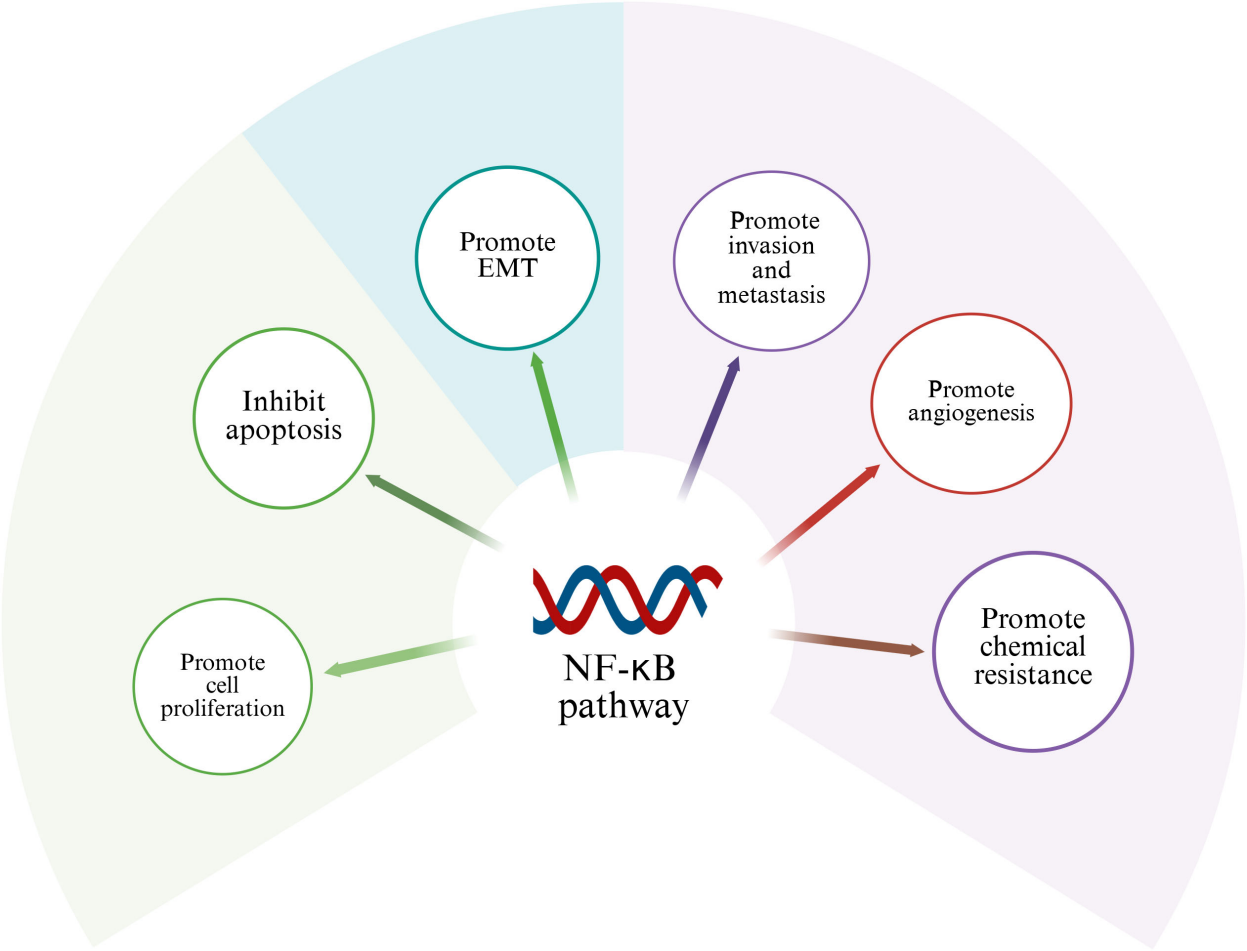
Role of NF-κB in osteosarcoma. Activation of NF-κB signaling pathway can promote cell proliferation, invasion, migration, tumor angiogenesis and chemical resistance of osteosarcoma.

NF-κB signaling network as a key regulatory hub in tumorigenesis, its abnormal activation is closely related to the multi-dimensional evolution of malignant tumors. At the level of cell cycle regulation, this pathway promotes the G1/S phase transition by transcriptional activation of the cyclin D1/CDK6 complex, and simultaneously upregulates cyclins such as cyclin A to maintain the persistence of proliferation. This dual regulatory mechanism provides a molecular basis for tumor cells to break through cell cycle checkpoints (51). It is notable that the anti-apoptotic function of NF-κB is signal-selective. In the TNF-α-mediated death receptor pathway, its nuclear translocation can induce the expression of survival factors such as c-FLIP and Bcl-xL, thereby constructing an apoptotic resistance barrier (52, 53). From the perspective of tumor biology, the carcinogenic effect of NF-κB presents multi-target characteristics: 1) It drives tumor angiogenesis by inducing the secretion of pro-angiogenic factors such as VEGF and IL-8; 2) Activate transcription factors to promote epithelial-mesenchymal transition (EMT); 3) Up-regulating drug drainage pumps (such as MDR1) and DNA repair enzymes enhances chemotherapy tolerance (12). Given the highly invasive and distant metastatic characteristics of osteosarcoma, the abnormal activation of NF-κB binds to specific DNA sequences existing in target genes, and regulates the proliferation, survival, angiogenesis, metastasis of cancer cells and the transcription of genes related to treatment resistance through spatiotemporal specificity (54) (**Table 1**). This chapter will discuss the role of abnormal activation of NF-κB in the above-mentioned pathological process (**Figure 2**).

**Table 1:** Role and mechanism of NF-κB pathway in OS.

Functional roles	Expression	Mechanism	References
Promotes cell proliferation	Upregulated	Shorten the multiplication cycle	(55, 56)
Inhibit apoptosis	Upregulated	By encoding the anti-apoptotic protein Bcl-2	(57)
Promote epithelial mesenchymal transformation	Upregulated	Enhance EGF activity	(58)
Promote invasion and metastasis	Upregulated	Inducing the expression of metastasis related proteins	(59)
Promote tumor angiogenesis	Upregulated	By regulating the expression of vascular endothelial growth factor (VEGF) and MMPs	(60)
Promote chemotherapy resistance	Upregulated	Promote the overexpression of P-glycoprotein (Pgp)	(61)

### 3.1 Role of NF-κB in the OS tumor microenvironment

Tumor microenvironment (TME) plays a crucial role in the occurrence, development and treatment resistance of various cancers such as osteosarcoma (62, 63). In osteosarcoma, there is a complex interaction between the NF-κB signaling pathway and the tumor microenvironment, which has an important impact on the occurrence and development of osteosarcoma, invasion and metastasis, and immune escape (64). Macrophages are one of the most abundant immune cells in the tumor microenvironment, and their polarization plays a key role in the genesis and development of tumors. In the osteosarcoma microenvironment, macrophages can be polarized into two main phenotypes, type M1 and type M2. M1 macrophages have pro-inflammatory and anti-tumor activities, and can secrete a large number of inflammatory cytokines to activate the immune response and directly kill tumor cells to inhibit tumor growth (65, 66). M2 macrophages have anti-inflammatory and pro-tumor activities, and can secrete immunosuppressive factors to promote the proliferation, migration and immune escape of tumor cells (67, 68). In the osteosarcoma microenvironment, the dominant phenotype shift to M2 is influenced by NF-κB regulators, including IL-10, transforming growth factor-β (TGF-β), and macrophage colony-stimulating factor (M-CSF) (69, 70). These M2-type macrophages can also secrete a variety of cytokines and growth factors, such as epidermal growth factor (EGF) and vascular endothelial growth factor (VEGF), which further promote the proliferation, migration and angiogenesis of tumor cells, forming a vicious cycle that promotes tumor development (71). In addition, NF-κB signal transduction can also enable immune cells such as regulatory T cells (Tregs) (72) and myeloid suppressor cells (MDSCs) (73) to form an immunosuppressive environment, secrete immunosuppressive factors, and inhibit the activity and function of CAR T cells. Thus, the microenvironment promotes immune escape of tumor cells, tumor angiogenesis, invasion and metastasis of tumor cells and treatment resistance (74, 75).

### 3.2 Promote cell proliferation

The autonomous growth mechanism of cancer cells often involves abnormal regulation of growth factor signaling pathways. Among them, the dysregulation of the NF-κB signaling pathway has been confirmed to be closely related to tumorigenesis and development. It is reported that the NF-κB signaling pathway is related to the inflammatory proliferation and differentiation of osteosarcoma cells (76). Basic research indicates that the NF-κB signal induces the loss of control of the cell cycle process by directly binding to the cyclin D1 promoter, thereby driving abnormal proliferation. Inhibition of NF-κB can block this effect, thereby inhibiting the proliferation of tumor cells (55, 56). Multiple mechanism studies have revealed the specific regulatory role of NF-κB in OS. In the MG63 cell model, the proliferation rate of the NF-κB high-expression group was significantly higher than that of the low-expression group. After inhibiting its activity through gene intervention, the cell proliferation ability could be reduced by more than 40% (28). This proliferative effect may be related to the inflammatory microenvironment it regulates. Studies have confirmed that the NF-κB pathway not only participates in the abnormal differentiation of osteosarcoma cells, but also promotes tumor growth by mediating the inflammatory factor network (76). It is worth noting that Huang’s team discovered the possibility of natural compound intervention - garnet derived from pomegranate juice can significantly reduce the proliferation rate of OS cells and disrupt their angiogenic ability by inhibiting NF-κB activity (77). It is worth noting that different research teams, through intervention methods such as gene silencing, small molecule inhibitors and natural compounds, have all observed a positive correlation between NF-κB inhibition and the decline in OS cell proliferation (78). The existing chain of evidence indicates that the proliferative effect of the NF-κB pathway in OS has multi-target characteristics: including regulating the expression of core cell cycle proteins (such as cyclin D1), maintaining the tumor inflammatory microenvironment, and affecting cell differentiation, etc. (79–81). This pleiotropic regulatory mechanism makes it a potential therapeutic target. Based on the above mechanism research, it is not difficult to see that the NF-κB signaling pathway plays an important regulatory role in the proliferation and growth of cancer cells in OS. Targeting the NF-κB pathway may provide a new strategy for the treatment of OS.

### 3.3 Inhibition of apoptosis

Apoptosis is a programmed cell death that plays a key role in maintaining tissue homeostasis and the response to tumor treatment. In malignant tumors such as OS, the efficacy of chemotherapy/radiotherapy largely depends on the activation of the apoptotic pathway, and tumor cells escape therapeutic pressure by establishing apoptotic resistance mechanisms. Anti-apoptosis is a common feature of cancer cells, which is related to the increased expression of anti-apoptotic factors such as Bcl-2 or Bcl-xL, or the decreased expression, inactivation or mutation of pro-apoptotic factors such as Foxo3a or p53 (82, 83). Studies have shown that this apoptotic imbalance involves the dynamic regulation of pro-apoptotic/anti-apoptotic factor expression, among which the NF-κB signaling pathway plays a central regulatory role (84, 85). From the perspective of molecular mechanisms NF-κB regulates the expression of Bcl-2 family members (such as Bcl-xL, A1/Bf1-1) and apoptosis inhibitor proteins (cIAP, TRAF) through direct transcription (57), inhibits the activity of pro-apoptotic factors (Foxo3a, p53) or induces their mutations, and regulates cell death-related proteases (such as caspase) 3) The activation threshold and other multiple dimensions are involved in the apoptosis resistance of OS (86, 87). This multi-target regulatory network makes NF-κB a key hub for maintaining the survival of tumor cells, and its continuous activation can lead to a decrease in the apoptosis rate of OS cells (88). Multiple intervention studies have confirmed the targeting ability of this pathway. Jiang’s team discovered that PPARα ubiquitination significantly increased the survival rate of OS cells by activating NF-κB, while pathway inhibition could increase the apoptosis rate (89). Furthermore, Chen et al. found that in OS, fluoxetine could inhibit anti-apoptotic protein (Bcl-2) and upregulate cleaved caspase-8, caspase-9 and caspase-3 to activate caspase-dependent apoptosis by suppressing the activity of the NF-κB tumor-causing signaling pathway (90). These studies indicate that the abnormal activation of the NF-κB pathway has an inhibitory effect on the apoptosis of cancer cells.

### 3.4 Promote epithelial mesenchymal transformation and invasion and metastasis

Epithelial-mesenchymal transition (EMT) refers to the transformation process in which adherent epithelial cells migrate to mesenchymal cells through molecular reprogramming. This plastic change plays a key role in the invasion and metastasis of malignant tumors (91). It is particularly worth noting that as a highly malignant tumor of mesenchymal origin, the characteristics of high early distant metastasis rate and poor prognosis in OS patients are closely related to the EMT process (92). Studies have confirmed that the NF-κB pathway drives the malignant progression of OS through a dual mechanism of directly activating the core transcription factors of EMT, inducing down-regulation of cell adhesion molecule expression and up-regulation of motion-related protein expression (77), and forming a positive feedback loop with growth factor signals (such as EGF) to continuously amplify the metastasis-promoting effect (93). Researches have shown that inhibiting EMT in osteosarcoma can significantly reduce the growth of osteosarcoma. As a core inducer of EMT, MT plays a crucial role in the metastasis and progression of many tumors (94, 95). Liu et al. (58) found that in osteosarcoma cells, EGF could induce the activation of the NF-κB pathway, and the activated NF-κB, in turn, enhanced the promoting effect of EGF on EMT. After using IκBα inhibitors to inhibit the activation of NF-κB, the morphology and viability of osteosarcoma cells can be significantly reversed, and cell migration can be weakened. This indicates that NF-κB plays a key role in EMT of osteosarcoma metastasis induced by EGF. Furthermore, NF-κB may also induce the expression of various metastasis-related molecules (such as intercellular adhesion molecule-1 (ICAM-1), vascular cell adhesion molecule-1 (VCAM-1), endothelial cell-leukocyte adhesion molecule-1 (ELAM-1), and the matrix metalloproteinase family (MMPs), etc.) Play a promoting role in the process of tumor invasion and metastasis. The above-mentioned molecules, as important mediators for cancer cells to migrate, penetrate the vascular wall and invade and metastasize target tissues, can respectively provide a favorable microenvironment for the dissemination and colonization of tumor cells through mediating cell adhesion, destroying the extracellular matrix structure and other pathways (12, 96–99). Studies have shown that in the osteosarcoma cell line U20S, the activation of the NF-κB signaling pathway can up-regulate key metastasis-promoting factors such as vascular endothelial growth factor (VEGF), MMP2 and MMP9. Among them, VEGF provides supporting conditions for the migration and invasion of cancer cells by promoting tumor angiogenesis, and MMP2/MMP9 by degrading the extracellular matrix. Experiments have confirmed that when the activity of this pathway is blocked by using the NF-κ B-specific inhibitor QNZ, the expression levels of the above-mentioned pro-metastasis related proteins significantly decrease, accompanied by a significant weakening of the migration and invasion abilities of tumor cells (100). Furthermore, multiple studies have further revealed the key role of the NF-κB signaling pathway in the progression of OS. Studies have shown that this pathway presents an abnormally activated state during the occurrence and development of OS. Activated NF-κB can significantly enhance the invasion ability of tumor cells and accelerate the metastasis process by promoting the secretion of MMPs (59). It is worth noting that Liao and his team (101) found through cell-level research that compared with normal bone cells, the expression level of the NF-κB signaling pathway in the OS cell line U20S was significantly increased, and its abnormally high expression state was positively correlated with the enhancement of the malignant phenotype of tumor cells (including invasion and metastasis ability). Functional verification experiments indicated that after specifically knockout the expression of NF-κB subunit using short hairpin RNA (shRNA), the invasion and metastasis abilities of osteosarcoma cells were significantly inhibited, suggesting that the continuous activation of this pathway is an important condition for maintaining the malignant behavior of tumor cells. Furthermore, multiple independent studies have confirmed that the abnormal activation of the NF-κB pathway has a universal carcinogenic promoting effect during the invasion and metastasis of OS cells (102–104).

Through the above research, it can be determined that the activation status of the NF-κB signaling pathway is closely related to the invasion and metastasis ability of osteosarcoma. Activated NF-κB provides necessary conditions for the invasion and migration of tumor cells by regulating the expression of downstream target genes. Therefore, targeting and inhibiting the abnormal activation of the NF-κB signaling pathway is expected to become a potential therapeutic strategy for blocking the malignant progression of OS and improving the prognosis of patients.

### 3.5 Promote angiogenesis

The development process of malignant tumors is jointly regulated by multiple molecular mechanisms, among which the activation of pro-angiogenic phenotypes is regarded as a key link in the continuous growth, invasion and metastasis of tumors. Neovascularization not only participates in the physiological remodeling of normal tissues, but also plays a core role in shaping the pathological microenvironment of various solid tumors such as OS (105). It is reported that the abnormal activation of the NF-κB signaling pathway can promote tumor angiogenesis through a dual regulatory mechanism of up-regulating the expression of vascular endothelial growth factor (VEGF) gene to enhance pro-vascular signaling and accelerating extracellular matrix remodeling by activating members of the MMPs family (106, 107). As the most important angiogenesis regulatory factor, VEGF can not only significantly increase capillary permeability, but also mediate the directional migration of endothelial tip cells by activating VEGFR2 receptors (108). In addition to degrading type IV collagen, MMPs can also release pro-angiogenic peptides by lysing matrix components such as laminectin. Their pro-endothelial proliferation effect shows obvious dose-dependent characteristics within a specific concentration range (109). In the osteosarcoma SaOS-2 cell model, Western blotting analysis revealed that the NF-κB signaling node was in a persistently activated state, and the expression level of its downstream effector molecule VEGF was significantly upregulated compared with normal bone cells. It is worth noting that after selectively blocking the nuclear translocation of NF-κB with small molecule inhibitors, the ability of tumor conditioned medium to induce vascular network formation decreases, suggesting that this pathway mediates tumor neovascularization by regulating the secretion of pro-angiogenic factors (80). Mechanism studies have shown that the abnormal activation of NF-κB not only directly promotes the biosynthesis of MMPs (especially MMP-2/9) through transcriptional regulation, but also creates three-dimensional spatial support for the proliferation and migration of endothelial cells by degrading type IV collagen and fibronectin in the basement membrane. This cascade reaction eventually drives vascular endothelial cells into an abnormal proliferation cycle, forming a functional tumor vascular network (60). In the tumor neovascularization regulatory network, in addition to VEGF and MMPs, there is also a cascade regulatory system coordinated by multiple factors. Pathological observations show that when the volume of solid tumors exceeds the vascular supply capacity, the hypoxic microenvironment triggers an increase in the stability of the HIF-1α subunit, and then forms HIF-1 heterodimers with transcriptional activity. This molecule can transcribe and activate pro-angiogenic mediators including platelet-derived growth factor (PDGF), transforming growth factor -β (TGF-β), and basic fibroblast growth factor (bFGF), thereby promoting OS angiogenesis (110–112). Further analysis revealed that these growth factors were not only potent inducers of angiogenesis, but also could activate the IKK kinase complex through ligand-receptor interactions, triggering the cascading amplification of the NF-κB signaling pathway. This bidirectional regulatory mechanism suggests that the abnormally activated NF-κB may act as a molecular hub, integrating the pro-vascular signals of factors such as PDGF/TGF-β/bFGF to form a continuously activated positive feedback loop in the OS. Although there are still gaps in the current research on the spatiotemporal dynamic regulation of vascular mimetic formation in OS by NF-κB, especially its relationship with angiogenic mimetic formation in tumor stem cells has not been clarified, the existing evidence has fully confirmed that this pathway drives pathological angiogenesis through a multi-target regulatory network.

### 3.6 Promoting chemical resistance

There are several mechanisms by which drug resistance develops in cancer chemotherapy, such as drug inactivation, apoptosis inhibition, enhanced DNA repair, and altered drug metabolism. One of the most critical mechanisms is regulation by ATP-binding box (ABC) proteins, a family of membrane transporters that excrete cytotoxic molecules, such as cell-lethal chemotherapy drugs (113). Researches have shown that NF-κB signaling plays a crucial role in chemical resistance (114). In chemical resistance, NF-κB activates up-regulated anti-apoptotic proteins, such as Bcl-2, Bcl-xL, and apoptotic protein inhibitors (cIAP). These proteins counteract key components of intrinsic and extrinsic apoptotic pathways and protect osteosarcoma cells from chemotherapy-induced apoptosis (115, 116). In addition, the activation of NF-κB regulates the expression of genes associated with drug resistance, such as multidrug resistance protein 1 (MDR1), which encodes P-glycoprotein (Pgp), a drug effector pump that reduces intracellular drug concentration by actively transporting chemotherapy drugs out of cells (117). In this regard, it has been suggested that inhibiting NF-κB signaling in osteosarcoma can effectively inhibit the overexpression of Pgp to prevent paclitaxel-induced multidrug resistance (61). This allows osteosarcoma cells to escape the cytotoxic effects of chemotherapy and continue to proliferate, leading to the development of chemotherapy resistance and tumor recurrence. In summary, abnormal activation of NF-κB signaling pathway in OS can regulate the proliferation and apoptosis of osteosarcoma cells, invasion, migration, chemical resistance to tumor angiogenesis, and tumor microenvironment.

## 4 The effect of crosstalk between NF-κB pathway and other signaling pathways on OS

**Figure 3:**
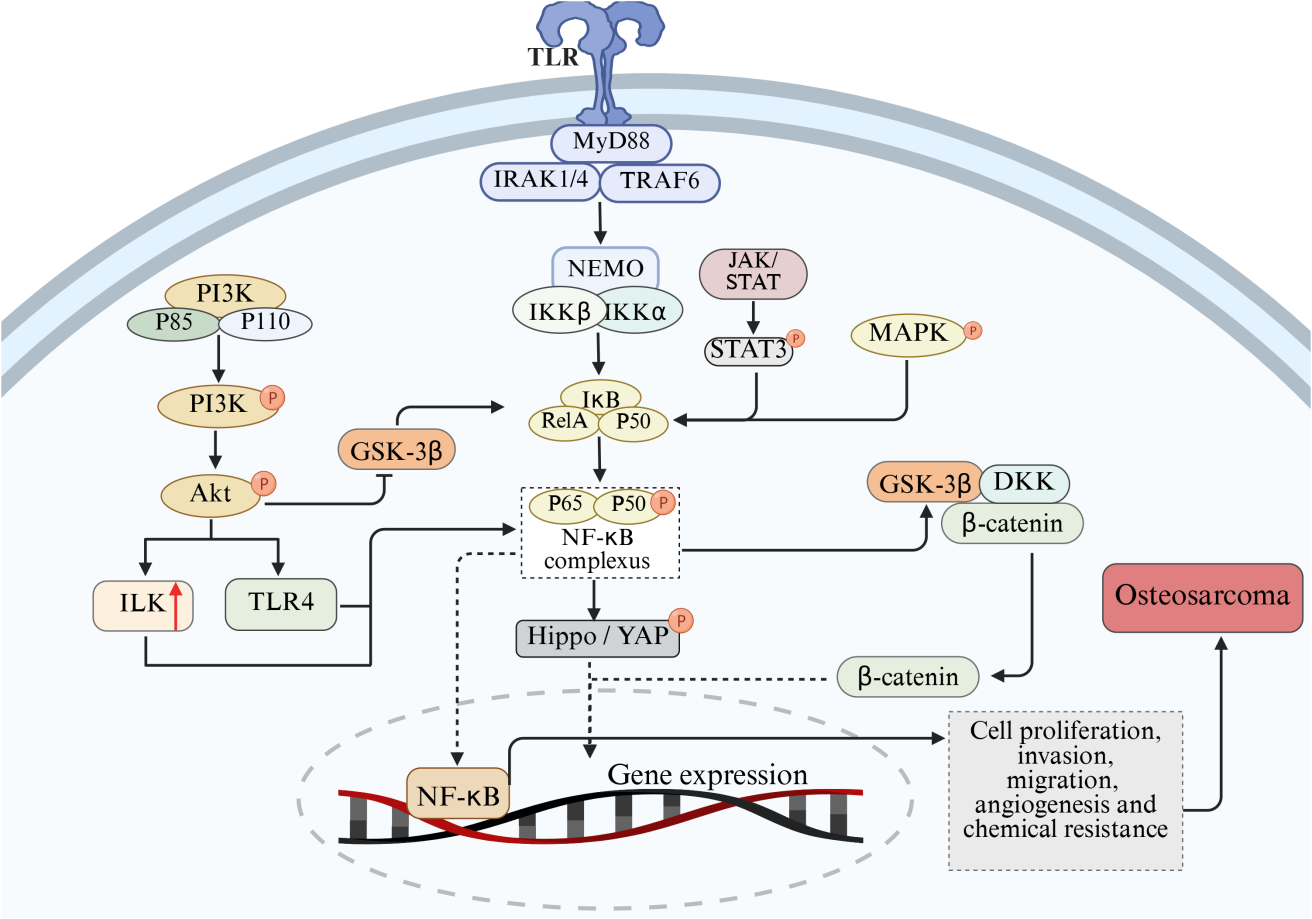
Mechanism of cross-talk between NF-κB and PI3K/Akt, Wnt/β-catenin, JAK/STAT, Hippo/YAP and MAPK pathways on the pathological process of OS.

In addition to the abnormal expression of NF-κB pathway, abnormal activation of other signaling pathways also play an important role in the pathogenesis of OS, such as phosphoinositol 3-kinase/protein kinase B (PI3K/Akt), Wnt/β-catenin and JAK/STAT signaling pathways. Signaling pathways interact, and enhancement of one signaling pathway may enhance or inhibit the other. In the process of tumor formation and development, NF-κB signaling pathway can directly or indirectly interact with other signaling pathways to regulate the pathophysiological processes of OS. This section describes the interaction of NF-κB with the PI3K/Akt, Wnt/β-catenin, JAK/STAT, and other signaling pathways in OS (**Figure 3**).

### 4.1 PI3K/Akt signaling pathway

The phosphatidylinositol 3-kinase/protein kinase B (PI3K/Akt) signaling pathway is an important signal transduction bridge connecting extracellular signals and cellular responses (118). PI3K is a large class of signal lipidases and a family of enzymes that phosphorylate the 3’-OH of the phosphatidylinositol inositol ring (119). Akt is an evolutionarily conserved serine protein kinase of the serine/threonine kinase family and is the central mediator in PI3K signaling (120). The PI3K/Akt signaling pathway is a highly conserved signal transduction network in eukaryotic cells, which can promote cell survival, cell growth and the cell cycle process (121, 122). Previous studies have found that the PI3K/Akt signaling pathway is a common activation pathway in human cancers, and it is believed that the dysregulation of this pathway can drive the occurrence and development of cancer and participate in the regulation of cancer pathological processes (123–125).

In malignant bone tumors represented by osteosarcoma, the PI3K/Akt and NF-κB signaling axes often show co-activation characteristics. Clinical sample analysis shows that approximately 65% of the cases simultaneously have enhanced phosphorylation at the Akt1 Ser473 site and RelA/p50 nuclear translocation phenomena (126, 127). There is evidence suggesting that there is an interaction between the PI3K/Ak signaling pathway and NF-κB (128, 129). These two pathways play important roles in the occurrence and development of OS by regulating the cell cycle, inhibiting apoptosis, promoting angiogenesis, enhancing metastasis and inducing chemotherapy resistance (130, 131). In the interactive network of the signal transduction cascade, NF-κB has been identified as the functional downstream effector of the PI3K/Akt pathway. Among them, the phosphorylation status of the Ser473/Thr308 dual-site of Akt directly regulates the nuclear-cytoplasm shuttling ability of the NF-κB transcriptional complex (132). Mechanism studies have shown that various pro-inflammatory cytokines (such as TNF-α, IL-6) can induce PDK1-dependent activation of Akt by activating the p85 regulatory subunit of PI3K, and thereby enhance the response sensitivity of the NF-κB signaling module through spatial conformational changes (133, 134). Functional experiments have confirmed that the PI3K-specific inhibitor LY294002 not only blocks the degradation of IκBα induced by IL-1β, but also retains it in the cytoplasm by inhibiting the nuclear localization signal (NLS) exposure of the p65-RelA allodimer (135). Akt accelerates the ubiquitination and degradation of IκBα by specifically phosphorylating the kinase domain of IKKα (Lys440 site). The released p50/p65 complex up-regulates the expression of metastasis-related genes such as MMP-9 and CXCR4 after entering the nucleus, ultimately promoting the migration ability of osteosarcoma cells (136–138). It is notable that PI3K/Akt can also activate integrin-linked kinase (ILK), and ILK can in turn activate the NF-κB pathway, thereby inducing EMT, angiogenesis, cell migration and invasion, and inhibiting apoptosis (139). Conversely, the overexpression of IκBα not only downregulates the cell transformation efficiency induced by Akt, but also leads to the weakening of mTORC1 signal output, confirming the existence of a metabolism-transcriptional coupling effect in the two pathways (140). Furthermore, glycogen synthase kinase-3 β (GSK-3β), as a key brake molecule of PI3K/Akt, its activity is inhibited by AKT-mediated Ser9 phosphorylation. Functional GSK-3β can antagonize NF-κB signaling through two mechanisms: enhancing the binding capacity of IκBα to p65 by phosphorylating Tyr42 and hindering the conformational adjustment of the binding domain of DNA by directly modifying the Ser468 residue of RelA (141–143).

### 4.2 Wnt/β-catenin signaling pathway

Wnt/β-catenin pathway is a family of proteins that play a key role in embryonic development and adult tissue homeostasis (144). Dysregulation of Wnt/β-catenin signaling often leads to a variety of serious diseases, including cancer and non-cancer diseases (145). Research have shown that there is a complex interactive regulatory relationship between the NF-κB signaling pathway and the Wnt/β-catenin signaling pathway in the development of osteosarcoma, and the two pathways interact to jointly affect the biological behavior of osteosarcoma cells, especially in the characteristics of tumor stem cells, tumor cell proliferation and metastasis (146). It was found that NF-κB can up-regulate the expression of Wnt ligand and promote the activation of Wnt/β-catenin signaling pathway. NF-κB can also directly interact with β-catenin to enhance the binding ability of β-catenin to TCF/LEF, thereby promoting the transcription of target genes (147). In addition, several studies have shown that the NF-κB and Wnt signaling pathways collaborate at multiple levels in different physiological and pathological contexts. IKKα and IKKβ are key activators of the NF-κB pathway, which regulate Wnt/β-catenin signaling activity in different ways (148). IKKα inhibitors block the expression of CCND1, a downstream Wnt gene, in mouse embryonic fibroblasts (147). Activation of NF-kB may increase the production of WNT-1, thereby triggering activation of Wnt/β-catenin and inducing tumor development (149).

### 4.3 JAK/STAT signaling pathway

The Janus kinase/Activator of Transcription protein (JAK/STAT) signaling pathway is an evolutionarily conserved cytokine signaling pathway, which not only controls the proliferation, survival and differentiation of stem cells, but also regulates calcium ion homeostasis, cell polarity and adult tissue homeostasis, especially in skeletal system diseases (150). Especially, the JAK2/STAT3 pathway is considered an important intracellular pathway within the JAK/STAT pathway and plays a crucial role in the occurrence and development of cancer (151). Research has shown that this pathway is involved in fundamental biological processes such as the proliferation, survival, invasion, and metastasis of tumor cells, and it also plays a role in the occurrence and progression of many malignancies (152, 153). NF-κB and STAT3 act as oncogenes, enhancing the metastasis potential of tumor cells and promoting tumor development and progression (154). There may be crosstalk between NF-κB and JAK/STAT signaling pathways in OS. Dephosphorylated STAT3 acts as an upstream signaling factor for NF-κB activation by binding to the IκB complex to promote NF-κB activation (155). STAT3 can also acetylate p65 in the nucleus, increasing the time it stays in the nucleus, thereby ensuring the constitutive activation of NF-κB. NF-κB and STAT-3 work together to control a common set of cytokine and chemokine coding genes that are involved in tumor development (156).

### 4.4 Other signal paths

In addition to the PI3K/AKT, Wnt/β-catenin and JAK/STAT signaling pathways, the NF-κB signaling pathway also has crosstalk mechanisms with several other signaling pathways, which play an important role in the occurrence and development of osteosarcoma and jointly regulate the biological behavior of osteosarcoma cells.

Hippo/YAP pathway plays a key role in organ development, tissue homeostasis maintenance and tumorigenesis and development (157). In osteosarcoma, Hippo/YAP pathway interacts with NF-κB signaling pathway. After NF-κB activation, YAP expression can be up-regulated, YAP nuclear translocation can be promoted, and YAP gene transcription can be promoted. In addition, the Hippo/YAP pathway also regulates the NF-κB signaling pathway. After entering the nucleus, YAP/TAZ can interact with NF-κB to synergistically regulate the expression of target genes (158). Some studies have shown that YAP/TAZ and NF-κB can jointly bind to the promoter region of certain genes, enhance the transcriptional activity of genes, and promote the proliferation, migration and invasion of osteosarcoma cells (159).

In osteosarcoma, there is a complex phosphorylation regulatory relationship between MAPK and NF-κB pathways (50). The MAPK pathway mainly includes three major signal transduction pathways: extracellular signal-regulated kinase (ERK), c-Jun amino-terminal kinase (JNK) and p38 mitogen-activated protein kinase (p38 MAPK), which play key roles in various biological processes such as cell proliferation, differentiation, apoptosis and stress response (160). Studies have shown that activation of the MAPK pathway can affect the activity of the NF-κB signaling pathway through phosphorylation in osteosarcoma cells (161). When cells are stimulated by growth factors, cytokines, or stress, the MAPK pathway is activated and ERK, JNK, or p38 MAPK is phosphorylated, which in turn activates several transcription factors and signaling molecules, including key molecules in the NF-κB signaling pathway. ERK phosphorylates IKKβ and promotes the phosphorylation and degradation of IκBα, thereby activating the NF-κB signaling pathway. JNK and p38 MAPK also regulate NF-κB activity through phosphorylation, which phosphorylates the p65 subunit and enhances the transcriptional activity of NF-κB. In turn, activation of the NF-κB signaling pathway can also affect the activity of the MAPK pathway. NF-κB can regulate the expression of some MAPK pathway related molecules, such as growth factor receptors, cytokines, etc., thereby indirectly affecting the activation of MAPK pathway. In addition, NF-κB can also interact with some transcription factors in the MAPK pathway to synergically regulate gene expression and influence the biological behavior of osteosarcoma cells (30).

## 5 Therapeutic intervention of NF-κB signaling pathway in osteosarcoma

**Figure 4:**
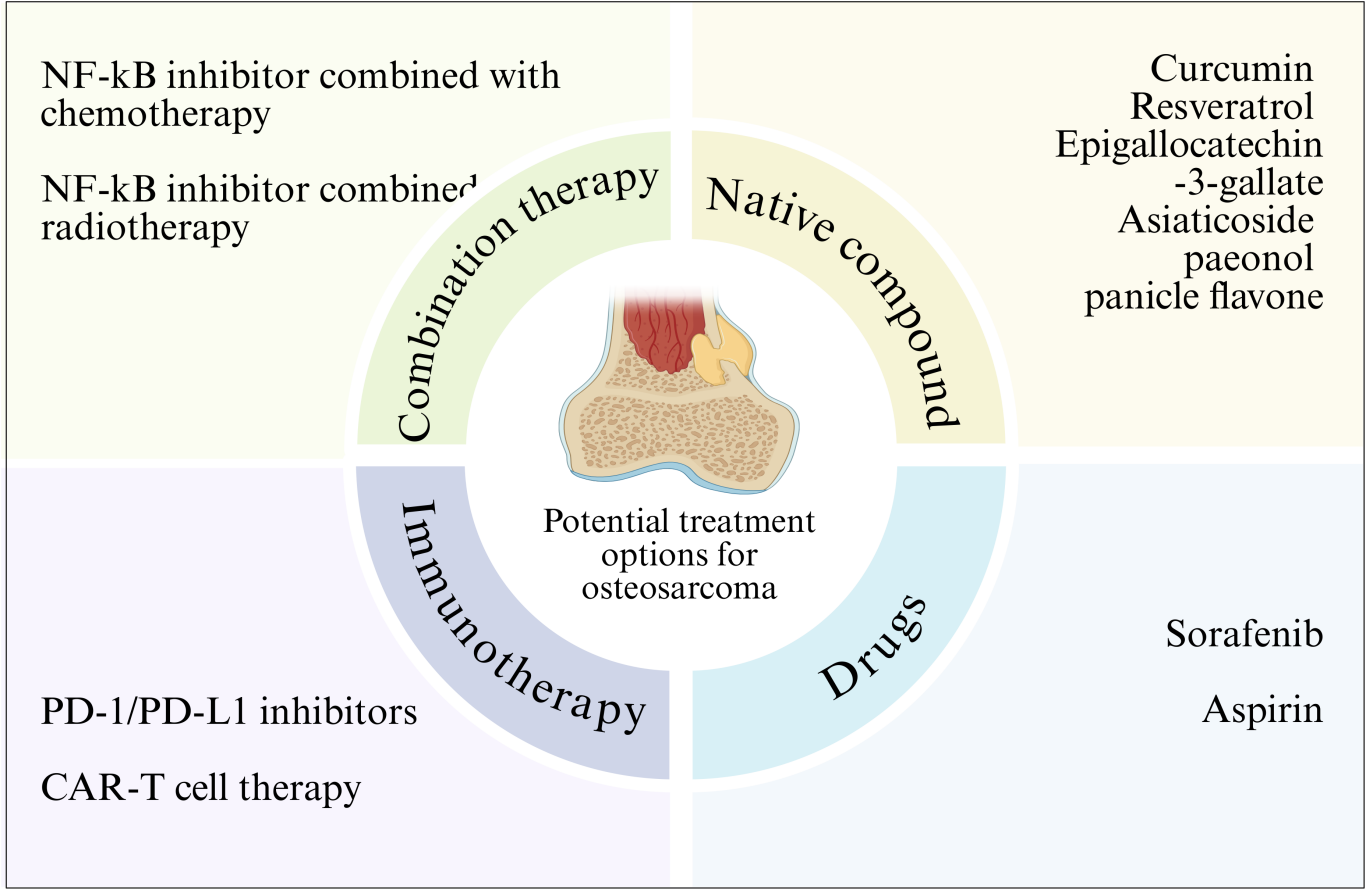
NF-κB signaling pathway related therapy in osteosarcoma. Various strategies for the treatment of osteosarcoma by targeting the NF-κB signaling pathway are outlined.

As an important pathway regulating cell growth, metabolism, survival, and chemotherapy resistance, targeting and inhibiting the NF-κB pathway may be a potential therapeutic approach for patients with OS (162). Several preclinical studies have confirmed that targeted therapy is a more effective treatment strategy for OS (163) (**Figure 4**). Therefore, targeted therapy of NF-κB pathway may be an important strategy for OS treatment.

### 5.1 Natural inhibitors

Given the importance of the NF-κB pathway in tumorigenesis, an increasing number of clinical studies have shown that different natural compounds can inhibit NF - κ B activity in cancer cells. Natural preparations or their derivatives have always been an attractive source of drugs. There are some natural compounds that have been shown to inhibit NF-κB, and the main compounds that have been approved for therapeutic use or are undergoing clinical trials are natural antioxidant compounds such as curcumin, briatrol and Epigallocatechin-3-gallate. Experimental studies have shown that these compounds show therapeutic promise in cancer, where these compounds can inhibit NF-κB activity and cancer cell growth, and induce cancer cell apoptosis (107, 164). This suggests that NF-κB may be a target for cancer prevention. In addition, synthetic antioxidants or NF-κB inhibitors also inhibit tumor growth by inhibiting NF-κB activity. Therefore, natural or synthetic antioxidants, which inactivate the activity of NF-κB, can act as molecular targeting agents against cancer. Targeting the NF-κB pathway is a promising approach for treating OS. Curcumin is a natural compound extracted from the rhizome of turmeric, which has various pharmacological activities such as anti-inflammatory, antioxidant and anticancer (165–168). Its anticancer effect has been confirmed in a variety of cancers, such as breast cancer, prostate cancer and colon cancer (169–173). Yang et al. confirmed that curcumin can enhance the polarization of macrophages and weaken the activation of NF-κB pathway, which has a protective effect on the osteoclast formation mediated by NF-κB receptor activator (RANK), thus inhibiting the development of osteosarcoma and repairing bone abnormalities (174). Resveratrol is a polyphenol chemical that has anti-cancer properties (175). Resveratrol has been reported to inhibit carcinogenesis by regulating intracellular signal transduction pathways. Previous studies have shown that resveratrol inhibits NF-κB activation by inhibiting the phosphorylation and subsequent degradation of IκBα (176). Research has found that in the osteosarcoma MG-63 cell line model, resveratrol can inhibit NF - κ B-mediated cytotoxicity of MG-63 cells, thereby suppressing cell proliferation, migration, and invasion, and activating cell apoptosis (177). In addition, asiaticoside (178), paeonol (30) and panicle flavone (100) can also significantly inhibit the mobility, migration and invasion of osteosarcoma cells by inhibiting the activity of NF-κB signaling pathway. Although natural compounds are less specific than targeted drugs, they have the advantage of low toxicity and can be used as an adjunct to other therapies.

### 5.2 Drugs

Several drugs have been shown to regulate the pathological process of OS by inhibiting NF-κB activity. For example, sorafenib is an oral multikinase inhibitor that has been shown to induce apoptosis through external and intrinsic signaling pathways, thereby inhibiting the growth of osteosarcoma cells (179).Wu et al. found that after treatment with sorafenib in osteosarcoma cells and animal models, NF-κB activity can be significantly inhibited to reduce the expression of anti-apoptotic protein and transfer-related protein (MMP-2, MMP-9) encoded by NF-κB target genes and reduce cell invasion of osteosarcoma (180). Aspirin inhibits NF-κB activity, and inhibition of the NF-κB pathway inhibits the growth of osteosarcoma and increases the sensitivity of osteosarcoma to chemotherapy *in vivo* and *in vitro* (181, 182). Liao et al. found in *in vivo* and *in vitro* experiments that aspirin treatment can effectively inhibit the activity of NF-κB pathway, thus significantly inhibiting the migration and invasion of osteosarcoma cells *in vitro* and reducing the incidence of metastasis of osteosarcoma xenograft tumors to the lung (183).

### 5.3 Immunotherapy

Immunotherapy as an emerging cancer treatment method, has shown great potential in the treatment of osteosarcoma, and the association between PD-1/PD-L1 inhibitors and the NF - κ B signaling pathway has attracted much attention. PD-1 (programmed death receptor 1) and PD-L1 (programmed death ligand 1) are immune checkpoint molecules that play a key role in tumor immune escape (184). In osteosarcoma, the expression of PD-L1 on the surface of tumor cells is up-regulated and binds to PD-1 on the surface of T cells to inhibit the activation and proliferation of T cells, thus enabling tumor cells to evade immune surveillance and attack of the body (185). Studies have shown that the NF-κB signaling pathway can regulate the expression of PD-L1 (186). Osteosarcoma cells activate the NF-κB signaling pathway when exposed to various stimuli, such as cytokines and inflammatory mediators. Activated NF-κB translocations enter the nucleus and bind to the promoter region of the PD-L1 gene, promoting its transcriptional expression. Therefore, inhibition of NF-κB signaling pathway can reduce the expression of PD-L1, enhance the immune activity of T cells, and improve the immunotherapy effect of osteosarcoma (187).

CAR-T cell therapy is a new type of immunotherapy, in which T cells are transformed into CAR-T cells that can specifically recognize tumor cell surface antigens through genetic engineering technology, and then injected back into the patient to achieve precise killing of tumor cells (188). In the treatment of osteosarcoma, CAR-T cell therapy has also shown some potential, with potential association with the NF-κB signaling pathway. Studies have shown that there are some specific antigens on the surface of osteosarcoma cells, such as Mesothelin and tumor-associated calcium signal transduction protein 2 (Trop2), which can be used as the target of CAR T cells. By constructing CAR T cells targeting these targets and injecting them back into osteosarcoma patients, tumor cells can be specifically recognized and killed (189).

### 5.4 Combination therapy

Combination therapy strategies have shown significant advantages in the treatment of osteosarcoma, and combining therapy targeting the NF-κB pathway with chemotherapy can play a synergistic role to improve the therapeutic effect (190). Chemotherapy is an important part of the comprehensive treatment of osteosarcoma, and commonly used chemotherapy drugs include methotrexate, doxorubicin and cisplatin (191). These chemotherapeutic drugs directly kill tumor cells through different mechanisms of action, such as inhibiting DNA synthesis and interfering with cell metabolism. However, chemotherapeutic drugs also have some limitations, such as the generation of drug resistance and toxic side effects on normal tissues. Studies have shown that targeting the NF-κB pathway can enhance the sensitivity of osteosarcoma cells to chemotherapy agents and overcome chemotherapy resistance (42). Abnormal activation of NF-κB signaling pathway is closely related to drug resistance of osteosarcoma cells. By inhibiting the NF-κB signaling pathway, the expression of drug-resistant related proteins, such as P-glycoprotein (P-gp), can be down-regulated to increase the concentration of chemotherapy drugs in tumor cells, thereby improving the efficacy of chemotherapy drugs (192). At the same time, therapy targeting the NF-κB pathway can also inhibit the proliferation, migration and invasion of tumor cells and induce apoptosis, which is complementary to the mechanism of action of chemotherapy drugs and plays a synergistic role (64).

Combining therapy targeting the NF-κB pathway with radiotherapy is also a promising combination therapy strategy. Radiotherapy is one of the important methods in the treatment of osteosarcoma. By irradiating tumor tissue with high energy radiation, the DNA structure of tumor cells is destroyed and apoptosis is induced. However, radiotherapy also has some limitations, such as radiotherapy resistance and damage to normal tissues (193). It has been found that targeting the NF-κB pathway can enhance the sensitivity of osteosarcoma cells to radiotherapy and improve the efficacy of radiotherapy. Abnormal activation of NF-κB signaling pathway is closely related to radiotherapy resistance. During radiotherapy, the NF-κB signaling pathway is activated after radiation irradiation, which up-regulates the expression of anti-apoptotic proteins, such as Bcl-2, XIAP, etc., while down-regulates the expression of pro-apoptotic proteins, resulting in the resistance of tumor cells to radiotherapy. Inhibition of NF-κB signaling pathway can reverse radiotherapy resistance and enhance the killing effect of radiotherapy on tumor cells. In addition, therapy targeting the NF-κB pathway can also reduce the damage to normal tissues caused by radiotherapy. During radiotherapy, normal tissues are also exposed to radiation, which activates the NF-κB signaling pathway, causing inflammation and tissue damage. Inhibition of NF-κB signaling pathway can reduce the secretion of inflammatory factors and reduce inflammatory response, thereby protecting normal tissues from radiation damage (64). These findings suggest that combination therapy targeting the NF-κB pathway can enhance the effect of radiotherapy and reduce the damage to normal tissue, providing a new idea for the treatment of osteosarcoma (**Table 2**).

**Table 2:** Inhibitors of NF-κB signaling pathway in osteosarcoma.

Drugs	Mechanisms		Reference
Natural inhibitor			
Curcumin		Inhibit the phosphorylation of NF-κB p65 and block the activation of NF-κB pathway→Induce cell apoptosis, inhibit metastasis and angiogenesis	(174)
Resveratrol		Inhibit p65 and IκBβ kinase →inhibit NF-κB pathway →Inhibit cell proliferation, migration and invasion, and activate cell apoptosis	(177)
Epigallocatechin-3-gallate		Inhibited the activation of IKK and phosphorylation of IkBα→inhibit NF-κB pathway →inhibits the proliferation of various cancer cells and induces apoptotic processes	(194)
Asiaticoside		Inhibit NF-κB signaling pathway→Inhibit the mobility, migration and invasiveness of osteosarcoma cells	(178)
Paeonol		Inhibit NF-κB signaling pathway→Inhibit the mobility, migration and invasiveness of osteosarcoma cells	(30)
Didemethyl-ginkgetin		Inhibit NF-κB signaling pathway→Inhibit the mobility, migration and invasiveness of osteosarcoma cells	(100)
Drugs			
Sorafenib		Inhibition of NF-κB activity→Reduction of anti-apoptotic proteins(Bcl-xl) and transfer-related proteins encoded by NF-κB target genes (MMP-2, MMP-9) →Inhibit migration and invasion	(180)
Aspirin		Inhibit NF-κB → Increase chemotherapy sensitivity of osteosarcoma, inhibit migration and invasion	(180, 181)
Immunotherapy			
PD-1/PD-L1 inhibitors		enhance the immune activity of T cells and improve the immunotherapy effect of osteosarcoma	(185)
CAR-T cell therapy		Specifically recognize and kill tumor cells	(189)

## 6 Clinical status and challenges

NF-κB signaling pathway is finely regulated as an intracellular signaling pathway, and its abnormal expression plays an important role in the occurrence and development of various malignant tumors, including OS. Structural activation of NF-κB is a novel hallmark of various tumor types, and many *in vitro* and animal models have shown that this pathway is involved in the regulation of multiple pathological processes such as cell proliferation, apoptosis, epithelial mesenchymal transformation, tumor angiogenesis, invasion and metastasis, and chemical drug resistance through complex molecular mechanisms. At present, the survival rate of patients has been improved under the multi-science and multi-mode therapy, but the prognosis of OS is not satisfactory. At present, there are still many challenges in the study of the role and mechanism of NF-κB signaling pathway in OS, which need to be further studied and explored.

### 6.1 Current research progress and challenges

Most existing preclinical reports suggest that the NF-κB pathway plays an important role in OS. At present, the studies on NF-κB and osteosarcoma are still at the level of *in vitro* and animal experiments. *In vitro* experiments, the activation of NF-κB signaling pathway is inhibited mainly by gene silencing technology, and the molecular mechanism of NF-κB signaling pathway in osteosarcoma is revealed. *In vivo* experiments, different doses of NF-κB pathway inhibitors were administered to observe tumor growth, metastasis, and animal survival to determine the optimal drug dose. Through such dose optimization study, it provides an important reference for clinical drug use, and helps to improve the safety and effectiveness of drug therapy.

Despite progress in preclinical studies on NF-κB signaling related treatment strategies, there are still many challenges in clinical trials that limit the widespread use and efficacy of these therapies in the clinical treatment of osteosarcoma. Although drugs targeting the NF-κB signaling pathway have shown some antitumor activity in preclinical studies, single-target drugs are often difficult to achieve the desired therapeutic effect in clinical trials. This is mainly because the development of osteosarcoma is a complex process involving the abnormal activation of multiple signaling pathways, and there are extensive interactions among the signaling pathways. Single targeting NF-κB signaling pathway is difficult to block all survival and proliferation signals of tumor cells, and tumor cells are easy to maintain growth and survival through compensatory effects of other signaling pathways. ​The problem of drug toxicity and drug resistance is also an urgent problem to be solved in clinical trials. Many drugs that target the NF-κB pathway inhibit the growth of tumor cells but also have toxic effects on normal cells. Some NF-κB inhibitors can cause damage to vital organs such as the liver and kidneys, limiting the dosage and duration of the drug. Resistance of osteosarcoma cells to targeted drugs is also a serious problem. Long-term use of drugs targeting the NF-κB pathway can cause tumor cells to gradually adapt to the effects of the drug and develop resistance by changing the activity or expression level of the signaling pathway. These drug toxicity and drug resistance problems have seriously affected the effectiveness of targeted therapy and the quality of life of patients. The difficulty of developing an individualized treatment plan is also one of the challenges facing clinical trials. Due to differences in gene expression, signaling pathway activity, and tumor microenvironment, osteosarcoma cells from different patients respond differently to drugs targeting the NF-κB pathway. How to make a personalized treatment plan according to the individual characteristics of patients is the key to improve the treatment effect. However, there is currently a lack of effective clinical methods to accurately predict a patient’s response to drugs. While some biomarkers have been found to correlate with drug efficacy, their predictive accuracy is not high enough to meet clinical needs. Although genetic testing technology can detect the genetic variation of patients’ tumor cells, there is a lack of in-depth research on how to select the appropriate therapeutic drugs and doses based on these genetic variation information. In addition, since OS tends to occur in children and adolescents, the NF-κB signaling pathway plays an important regulatory role in normal growth and development. Inhibition of NF-κB signaling pathway may interfere with normal growth and development in pediatric patients. In bone development, the NF-κB signaling pathway is involved in the differentiation and functional regulation of osteoblasts and osteoclasts. Inhibition of NF-κB may affect the activity of osteoblasts, resulting in bone mass loss, bone retardation and other problems. In terms of immune system development, the NF-κB signaling pathway is also critical for immune cell development and function. Inhibition of NF-κB may lead to abnormal differentiation and maturation of immune cells, affecting the normal function of the immune system and making pediatric patients more susceptible to infections. Therefore, it is necessary to further strengthen the research on individual differences in osteosarcoma and develop more effective predictive indicators and treatment strategies to achieve accurate treatment of osteosarcoma.

### 6.2 Future research direction

The transformation of basic research into clinical practice is a key step in achieving effective treatment of osteosarcoma, which still faces many challenges and needs to make progress in several key breakthrough points. In view of the bottleneck of efficacy of single-target drugs, the development of high-efficiency and low-toxicity combined therapy drugs is one of the key breakthrough points. Through multi-omics integrated analysis, the molecular characteristics of osteosarcoma cells and the regulatory mechanism of signaling pathways were fully revealed, and patients sensitive to combination therapy were screened out to achieve personalized treatment. Use AI-assisted drug design to accelerate the development process of combination therapy drugs and improve the specificity and efficacy of drugs. Artificial intelligence technology can conduct virtual screening of a large number of compounds and quickly discover drug molecules with potential combination therapy activity, providing new ideas for the development of combination therapy drugs.

To solve the problem of drug toxicity and drug resistance is also an important breakthrough point in the transformation of basic research into clinical practice. The mechanism of drug toxicity was studied deeply, and the toxicity of drugs to normal cells was reduced by optimizing the structure and improving the drug delivery system. In view of the problem of drug resistance, the mechanism of drug resistance is studied, and new strategies to overcome drug resistance are developed. The tumor microenvironment response characteristics of intelligent carrier system provide a new way to solve the problem of drug toxicity and drug resistance. A Ph-responsive polymer based nanocarrier loaded with NF-κB inhibitors was designed. *In vitro* experiments, the nanocarrier can rapidly release NF-κB inhibitors under acidic conditions, and effectively inhibit the proliferation and migration of osteosarcoma cells. Through this intelligent carrier system, the precise release of drugs can be achieved, the toxicity of drugs to normal cells can be reduced, and the efficacy of drugs can be improved. To study the regulatory mechanisms of drug-resistant signaling pathways, develop inhibitors targeting drug-resistant pathways, and use them in combination with drugs targeting the NF-κB pathway to overcome drug resistance.

Establishing effective individualized treatment plans is the core breakthrough point for translating basic research into clinical practice. Through the screening and verification of biomarkers, the patients’ response to treatment can be accurately predicted, and the basis for individualized treatment can be provided. Combined with the genetic characteristics of patients, tumor microenvironment and other factors, to develop a personalized treatment plan. In osteosarcoma studies, it has been found that some biomarkers related to the NF-κB signaling pathway, such as the nuclear expression level of the NF-κB subunit and the expression level of target genes such as Cyclin D1 and VEGF, are closely related to patient prognosis and response to therapy. By detecting these biomarkers, patients sensitive to targeting the NF-κB pathway can be screened for individualized treatment. Machine learning algorithms are used to analyze patients’ clinical data, genetic data and tumor microenvironment data to establish a personalized treatment model and provide decision support for doctors to make treatment plans.

## 7 Conclusion

NF-κB signaling pathway is an intracellular signaling pathway that is finely regulated, and abnormal expression of this pathway plays a crucial role in the occurrence and development of a variety of malignant tumors, including OS. The structural activation of NF-κB is a novel marker of OS, and it has been demonstrated *in vitro* and in animal models that this pathway is involved in the regulation of tumor cell proliferation, apoptosis, epithelial-mesenchymal transformation, tumor angiogenesis, invasion and metastasis, tumor angiogenesis, and tumor cell drug resistance through complex molecular mechanisms. Based on the role of NF-κB pathway in the development of OS, we suggest that this pathway may be a potential target for OS therapy. Targeting or manipulating the expression or function of the relevant NF-κB signaling pathway may be an innovative approach to treating OS and a potential target for cancer drug development.
